# Understanding the Influence of Consumers’ Perceived Value on Energy-Saving Products Purchase Intention

**DOI:** 10.3389/fpsyg.2021.640376

**Published:** 2022-01-31

**Authors:** Biao Luo, Liru Li, Ying Sun

**Affiliations:** ^1^School of Management, Hefei University of Technology, Hefei, China; ^2^School of Management, University of Science and Technology of China, Hefei, China; ^3^International School of Economics and Management, University of Beijing Technology and Business University, Beijing, China

**Keywords:** theory of consumption value, green experience, appraisal-emotional response-coping theory, energy-saving products, perceived value (PV)

## Abstract

Since rapid economic growth has led to the overuse of natural resources and environmental degradation, increasing attention has been paid to environmental problems. This study aims to explore the relationship between consumers’ perceived value and satisfaction, and energy-saving products purchase intention was investigated using appraisal-emotional response-coping theory. Moreover, this study further investigates these relationships in different consumer groups. In total, 399 questionnaires were collected online and offline, and results though structural equation modeling analysis show that functional, emotional, conditional, and green value have a positive effect on consumer satisfaction and thereby promote the intention to purchase energy-saving products. However, social value is not significant for consumer satisfaction. Perceived value influences consumer satisfaction and varies among different consumers according to the results of multigroup structural equation modeling analysis. These results have practical significance for the design and marketing of energy-saving products.

## Introduction

In recent years, with global warming gradually becoming the focus of public attention, people have begun to realize the significance of the environment and the impact of consumption on the environment (Khan and Mohsin, 2017; Strielkowski et al., 2019). Previous studies have pointed out that energy consumption leads to energy shortages and environmental problems (Appiah, 2018). It is of great significance to study consumers’ energy-saving purchase intention in China since China is the largest energy consume country in the world. Large energy consumption has brought a lot of carbon emissions and other toxic gases, which may harm the environment and personal health. Energy shortages and environmental problems cause people’s living environments to suffer severe challenges. Increasing concerns about environment lead consumers to turn their preferences to energy-saving products and services (Luchs et al., 2010; Kotler, 2011; Song et al., 2019). Thus, considering the negative impact and large energy-saving potential in China, several measures and related studies should be taken to reduce energy consumption and promote energy-saving products behavior.

Many existing studies have studied consumers’ purchase behavior of energy-saving products from economic-oriented perspective or technological-oriented perspective (Ma et al., 2011; Ha and Janda, 2012). For example, measures based on financial incentives can only be effective for a short time (Clune and Zehnder, 2020), but unless these financial measures last for a long time, consumers will not continue energy-saving products behavior (Wang et al., 2018c). Taking into account the limitations of the economic-oriented and technological-oriented perspectives, scholars have increasingly realized that a psychological-oriented perspective is of great significance to purchasing energy-saving products (Shi et al., 2017; Zheng and Liu, 2019). The perceived value of energy-saving products has a great value in improving consumers’ purchase of energy-saving products and reducing energy consumption, but the path between them is not clear yet. This article explores the perceived value dimension of energy-saving products and consumer purchase behavior based on the appraisal-emotional response-coping theory while considering boundary conditions (Sheth et al., 1991).

As mention above, previous research on perceived value and purchase behavior has found several contradictory effects among those constructs (Lin and Huang, 2012; Khan and Mohsin, 2017). This study addresses the inconsistency between perceived value and purchasing behavior. When establishing a model of consumer perceived value influences green purchase, we should not neglect the influence of consumer characteristics such as green purchase experience, gender, and income (Papista and Krystallis, 2013; Sun and Wang, 2019; Sun et al., 2021). Because consumers’ behavior will change with the purchasing experience they gain (Heilman et al., 2000). The use of energy-saving products may change consumers’ perceptions and attitudes. Therefore, consumers’ perception about buying energy-saving products for the first time may influence their subsequent decisions. Despite these changes, few studies in the green field have conducted analyses of perceptions related to energy-saving product use and decision-making after use. In addition, few scholars have analyzed the behavior of green consumers when they obtain experience. In other types of consumption, customers’ behavior is not necessarily stable over time (Jiang and Rosenbloom, 2005) because they will change their ideas as a result of their past purchasing experience. When consumers repeat their purchase behavior many times, they will become more aware of their real needs and form good purchase habits (Liao et al., 2006). Similarly, consumers of energy-saving products will be familiar with the characteristics of energy-saving products, pay attention to certain details of shopping, and ignore some aspects that may be important in the early stage. In addition, the demographic variables of consumers also show different consumption tendencies in the field of energy-saving consumption. For example, although male consumers have higher purchase attitudes toward energy-saving products, female consumers are more likely to make green purchase decisions (Sun and Wang, 2019). Moreover, the consumers with different income levels have also become important factors in the purchase of energy-saving products. The relationship between the perceived value and the satisfaction of purchasing energy-saving products will also have different effects among those subgroups. These factors should be discussed in depth in promoting the purchase strategy of energy-saving products in modern market segments. Therefore, this study addresses the research gap by empirically examining the impact of consumers with different green experiences, gender, and household income.

In view of the above, this study provides an integrated model to better understand purchasing intention toward energy-saving products based on the appraisal-emotional response-coping theory (Bagozzi, 1992). The perceived value of energy-saving products leads to an emotive state of satisfaction (emotional response), which ultimately leads to behavioral intention (coping response) (Kim et al., 2013). The aims of this article are to address the following two issues: (1) to analyze the perceived value of energy-saving products and which factors influence consumer satisfaction and thus influence their purchase intention in China; and (2) to explore the moderating effect of green experience, gender, and income. More specifically, each perceived value represents different levels of customers’ perception of energy-saving appliances. Customer satisfaction with the overall impression of energy-saving products is influenced by these dimensions. This study proposes that the influence of perceived value on satisfaction varies among different consumers. We carried out this analysis in China and distinguished six groups in the sample: (1) potential green customers, who are considering to make their first purchasing of energy-saving products; (2) experienced customers, who have purchased at least one energy-saving product; (3) male subgroups; (4) female subgroups; (5) low-income subgroups; and (6) high-income subgroups.

We believe that this research makes several contributions to the literature. First, this study has deepened the understanding of consumer intention for energy-saving products from psychological-oriented perspective and expands the study on energy-saving purchase behavior. Moreover, to better understand the value of energy-saving products, this study also stretches the theory of consumption value for energy-saving products. According to the green characteristics of energy-saving products, this study addresses green value, which makes the perceived value of energy-saving products more comprehensive. Finally, exploring the contingent effects of green experience, gender, and income on the relationship between consumer’s perceived value and energy-saving purchase intention, and providing a more comprehensive understanding of the intention to purchase energy-saving products. Consumers with green experience are familiar with energy-saving products and pay attention to green value, while potential consumers pay more attention to conditional value.

## Conceptual Framework and Hypotheses

### Theoretical Background

The existing literature on energy-saving consumption behavior research perspectives are mainly divided into three categories: economic-oriented perspective, technological-oriented perspective, and psychological-oriented perspective (Loock et al., 2013; Wang et al., 2018d). Economic-oriented scholars believe that rational energy-saving consumption is to obtain the greatest benefits at the lowest cost (Zhou and Yang, 2016). Then, economic measures such as price or fiscal incentives can promote energy-saving consumption behavior (Wang et al., 2018c). However, many studies have shown that the effects of these measures are not as good as imagined, because social factors such as habits and emotions will affect the assumption of rational people (Loock et al., 2013). Technology-oriented scholars believe that through the research and development of energy-saving technologies, the energy and environmental pressure brought by household energy use can be significantly alleviated (Zhou and Yang, 2016). However, Nilsson et al. (2014) found that after significant improvements in energy-saving technologies and energy-efficiency technologies, household electricity consumption not only did not decrease compared with the same period, but increased. Psychological behavior-oriented scholars believe that energy consumption behavior is affected by psychological factors such as attitudes, emotions, and environmental awareness (Zhou and Yang, 2016; Ding et al., 2017). The psychological-oriented perspective aims to achieve the goal of green environmental protection by emphasizing some psychological factors (such as attitudes, social norms, and environmental concern to promote the purchase of energy-saving products) (Fornara et al., 2016; Ruangkanjanases et al., 2020). Considering that residents’ energy consumption is caused by individual’s daily behavior, this study believes that it seems more reasonable to study energy-saving behavior from the perspective of psychological behavior (Wang et al., 2018c).

In the field of psychological-oriented perspective, we focus on perceived value because perceived value is the main driving force for consumers to generate purchase intention. We assume that environmentally friendly consumer behavior should be reflected in a more realistic selection context, and consumers must weigh their preferences for the benefits and costs of different products when evaluating energy-saving products (Papista and Krystallis, 2013). This article uses the customer value to try to identify the various factors that motivate or hinder consumers from adopting energy-saving products.

#### The Theory of Perceived Value

We highlight the definition of perceived value by Zeithaml (1988), the most universally accepted definition. He proposed that perceived value is “the consumer’s overall assessment of the utility which is based on perceptions between gain and loss.” Woodruff (1997) further extended the definition of perceived value, arguing that customer value is customers’ preference and evaluation of the product attributes and its utility that contribute to or hinder their achievement of the target in specific contexts; this extension has been accepted by many scholars.

As perceived value has received growing attention, a multidimensional construct to conceptualize perceived value was unanimously approved (Sheth et al., 1991; Sweeney and Soutar, 2001). Referred to the research methods of Reyes-Menendez et al. (2018, 2020), this study reviews the previous research on perceived value, as shown in Table 1. The multidimensional structure of perceived value has attracted the attention of scholars, but no consensus has been reached in the literature about the number of relevant dimensions (Gallarza, 2011). In the current research, Sheth et al. (1991) put forward the theory of perceived value, which classify perceived value into five categories including functional, emotional, social, epistemic, and conditional value. Perceived value theory is widely used because these categories are comprehensive and align with the consumer perspective (Biswas and Roy, 2015). Among them, epistemic value is defined as “the perceived utility derived from a product or service to arouse curiosity” (Sheth et al., 1991). Since consumers are very familiar with household appliances and the products cannot inspire their curiosity, epistemic value is not considered in this study. Meanwhile, previous studies have shown that other dimensions could be added or substituted depending on the actual condition (Koller et al., 2011; Hur et al., 2013; Wang et al., 2018a). Energy-saving, unlike ordinary products, have the special feature of environmental protection. In the green field, the most prominent feature of green products is green, but the green value dimension is rarely paid attention to. Given the unique attributes of household appliances (e.g., power consumption and environmental impact), green value is incorporated into the theory of perceived value. Therefore, this study uses the theory of perceived value and studies five dimensions of functional value, emotional value, social value, conditional value, and green value.

**TABLE 1:** Selected studies on dimensions of perceived value.

Dimensions of perceived value	Condition	Theme	References
Product quality, value for money, emotional value, and social value	Retailing	How PERVAL dimensions of value affect customers’ loyalty, through both cognitive and affective satisfaction.	Gallarza et al., 2016
Functional value, social value, emotional value, conditional value, and epistemic value	Green products	the consumer choice behavior for green products	Khan and Mohsin, 2017
Functional value, emotional value, social value	Smartphone brands	What drives consumers’ loyalty to smartphone brands	Yeh et al., 2016
Functional value quality, functional value price, social value identity, social value responsibility, emotional value, and conditional value	Green electronics	Consumer choice behavior regarding green electronics	Danish et al., 2019
Functional, conditional, green, and social values	Adoption of bicycle sharing	The impact of perceived value on consumer adoption intentions, and assesses the moderating effects of social and personal attitudes toward environmental behavior on perceived value-adoption intention relationships.	Wang et al., 2018d
Functional value, economic value, emotional value, green value, social value, epistemic value, and ethical value	Sharing economy setting of Airbnb	The co-creation of customer-perceived value between customers and service providers in the sharing economy setting of Airbnb	Jiang et al., 2019

#### The Appraisal-Emotional Response-Coping Theory

Although the appraisal-emotional response-coping theory is widely used in the service industry (Zourrig et al., 2009), this article intends to test this relationship in the green field. This theory explains the behavior process, that is, specific emotions are generated based on different evaluations and subsequently lead to a variety of coping responses. Bagozzi (1992) proposed that the appraisal is an evaluation of outcome desire. When an individual’s requirements are not met, there will be an outcome desire conflict; when the requirements are met, the outcome desire will be content. These outcome desires will produce positive or negative emotional responses. The positive reaction will have the behavioral intention to enhance this result, while the negative reaction will have the behavioral intention to weaken the conflict.

Scholars have found that perceived value is closely related to cognitive appraisals according to theory (Bagozzi, 1992). Therefore, it leads to the emotive state of satisfaction (emotional response), which ultimately leads to behavioral intention (coping response) (Kim et al., 2013). Customers are more easily satisfied when they perceive more value in a product (Jiang et al., 2016), and satisfied consumers tend to buy the product (Yang and Peterson, 2004).

### Hypothesis Development

#### Perceived Value and Satisfaction

The definition of satisfaction in the existing literature is different. However, customer satisfaction can be described according to two perspectives, that is, transaction and cumulative perspectives (Yang and Peterson, 2004). From the transaction perspective, customer satisfaction is defined as an emotional response after a specific purchase (Oliver, 1993). From the cumulative perspective, customer satisfaction is evaluated after a period of consumption associated with specific products or firms (Ram and Wu, 2016). Compared to transactional customer satisfaction, overall satisfaction can illustrate consumers’ stable sentiment. In this study, satisfaction is a judgment of the products and services or the extent to which the consumers’ expectations are fulfilled (Oliver, 2014). The role of perceived value has been demonstrated as an antecedent of customer satisfaction in different contexts in previous research (Woodruff, 1997; Hur et al., 2013; Jiang et al., 2019). Different value dimensions have different roles in user decisions (Deng et al., 2010). Based on the first part of appraisal-emotional response-coping theory, satisfactions (specific emotions) are generated based on different perceived values (evaluations).

Functional value is considered as the main antecedent of consumer choice (Wang et al., 2018d). The functional value of energy-saving products is the perceived utility derived from the specific attributes of products such as service life, price, and quality. When deciding whether to purchase energy-saving products, consumers evaluate price and quality closely (Bei and Simpson, 1995). Quality and price are deemed to be the main drivers of consumer choice behavior in energy-saving product purchase (Sheth et al., 1991; Bei and Simpson, 1995) decisions. Although the selling price of energy-saving products is more expensive than ordinary products, these products are more energy-saving in the use process. In the long run, they are cost-effective and economical, and also of good quality. If consumers perceive that energy-saving appliances are high quality and the price is acceptable (i.e., cost-effective), they will be satisfied with the appliances and have higher intention to purchase them. Thus, the following hypothesis is proposed and tested:

H1a: Functional value is positively related to satisfaction with energy-saving products.

Sheth et al. (1991) defined emotional value as “the perceived utility derived from a product arousing feeling or affective states.” Emotional value for energy-saving products is the perceived utility acquired from the product’s ability to bring a pleasant and comfortable feeling. Although consumers may not deliberately pursue emotional interests in the consumption process, the positive feelings generated in the consumption process will play a key role on the subconscious level of decision-making (Hur et al., 2013). Consumers who buy energy-saving products may think that they make their own contribution to environmental protection, save energy, and enhance their green beliefs (Lin and Huang, 2012). Consumers with positive emotional value for energy-saving products will have a good impression of such products and feel satisfied. Therefore, we propose the following hypothesis:

H1b: Emotional value is positively related to satisfaction with energy-saving products.

Sheth et al. (1991) defined social value as “the perceived utility acquired from an alternative’s association with one or more specific social groups.” Social value for energy-saving products is the perceived utility acquired from enhancing a customer’s self-image and obtaining social acceptance. Consumers consider the opinions of people around them when purchasing products (Suki, 2016). Energy-saving products are capable of slowing resource consumption. Consumers may gain social value from energy-saving products, as they may be considered to be environmentalists and socially responsible. Social responsibility can also stimulate people’s environmental behavior (Biswas and Roy, 2015). When purchasing energy-saving products, consumers will obtain not only social recognition but also the respect of others. As a result, consumers will easily feel satisfied because of extra social respect when they purchase energy-saving products. Therefore, we propose the following hypothesis and test:

H1c: Social value is positively related to satisfaction with energy-saving products.

Sheth et al. (1991) defined conditional value as “the perceived utility derived from a specific situation or circumstances.” Conditional value for energy-saving products is the perceived utility derived from discounts or promotions. Previous studies (Laaksonen, 1993; Lin and Huang, 2012) have shown that consumers’ choice behavior is related to their personal situation. In China, due to the government’s policy of vigorously promoting environmental protection, the purchase of energy-saving household appliances will provide consumers with government subsidies, that is, consumers will be given a discount for the purchase of energy-saving household appliances. Meanwhile, there is barely a discount for ordinary household appliances. Consumers always tend to buy promotional products (Aydin and Ziya, 2008). Thus, consumers will feel satisfied because of the discounts when they select energy-saving products. Therefore, we propose the following hypothesis:

H1d: Conditional value is positively related to satisfaction with energy-saving products.

Green value is the perceived utility derived from consumers’ environmental desires, sustainable expectations, and green needs, such as reducing environmental pollution and saving power (Patterson and Spreng, 1997; Jiang et al., 2019). Research shows that the products’ degree of greenness may affect the behavior of different types of consumers (Wang et al., 2018e). When consumers purchase energy-saving products, they may consider how much the product impacts the environment and whether the product harms their health. Consumers who understand environmental problems and the green value of energy-saving products will easily have a better impression of energy-saving products and thus form a better satisfaction of them. Therefore, we propose the following hypothesis:

H1e: Green value is positively related to satisfaction with energy-saving products.

#### Satisfaction, Purchase Intention

Customers usually make a purchase decision after evaluating whether their previous experience of using a product is satisfactory (Kim et al., 2013; Prebensen et al., 2014; Ali et al., 2016). Therefore, satisfaction is the source of the company’s competitive advantage (Oliver, 1999). Meanwhile, it is also significant to comprehend customers’ purchase intention because their intention is usually reflected in their actions (Bai et al., 2008). Most scholars who investigate the satisfaction and behavioral intention link have shown that satisfaction is a strong antecedent of behavioral intention to purchase various types of green products, such as green detergent brands (Papista et al., 2018), hybrid cars (Hur et al., 2013), and eco-friendly cruises (Han et al., 2018). When consumers are satisfied with a product, their desire to buy and experience that product usually increases. In contrast, when consumers are dissatisfied with a product, their positive intentions and desires to buy and experience that product will be reduced (Han and Ryu, 2012). Drawing on the second part of appraisal-emotional response-coping theory, if consumers are satisfied with energy-saving products, they will be motivated to purchase such products. Therefore, we propose the following hypothesis:

H2: Purchase intention is positively influenced by consumer satisfaction.

#### The Moderating Effect of Green Experience, Gender, and Income

Green experience is the experience of consumers’ past purchase of green products. The relationship between perceived value and purchase behavior has different results in the study of energy-saving products (Lin and Huang, 2012; Khan and Mohsin, 2017). Consumer purchasing behavior is largely influenced by historical behavior (Schwabe et al., 2018), which may be the main reason for the inconsistent results. When consumers perceived same value of energy-saving products, consumers who have purchased energy-saving products will be more convinced of the utility because of their previous purchase experience, and they are more likely to get higher satisfaction (Zhou and Zhang, 2019). Conversely, consumers who have never purchased similar products have more worries, such as worrying about the quality of the products and doubt about them, which will reduce satisfaction. Thus, we propose the following hypothesis:

H3: The effect of the perceived value of energy-saving products on satisfaction is moderated by the consumer’s green experience; this influence is significantly greater among potential customers than experienced customers.

Gender differences in decision-making and information processing lead to a variety of buying behaviors (Sun and Wang, 2019). Scholars have proved that women and men have significant differences in their physiological and social needs. For example, compared with men, women are more social, emotional, and caring about others (altruism). When perceiving the same perceived value, women are more tolerant of energy-saving products and more likely to have higher satisfaction. Also, individuals’ income significantly affects their perception of perceived value (Shen et al., 2021). The income will affect individual price sensitivity and makes low-income groups have more product search behavior, thereby reducing the risk of purchase. Thus, consumers are more likely to perceive the value of the product, which will also produce higher satisfaction. Therefore, the income significantly moderates the relationship between perceived value and consumer satisfaction. Thus, we propose the following hypotheses:

H4: The effect of the perceived value of energy-saving products on satisfaction is moderated by gender; this influence is significantly greater among female group.
H5: The effect of the perceived value of energy-saving products on satisfaction is moderated by income; this influence is significantly greater among low-income group.

**FIGURE 1:**
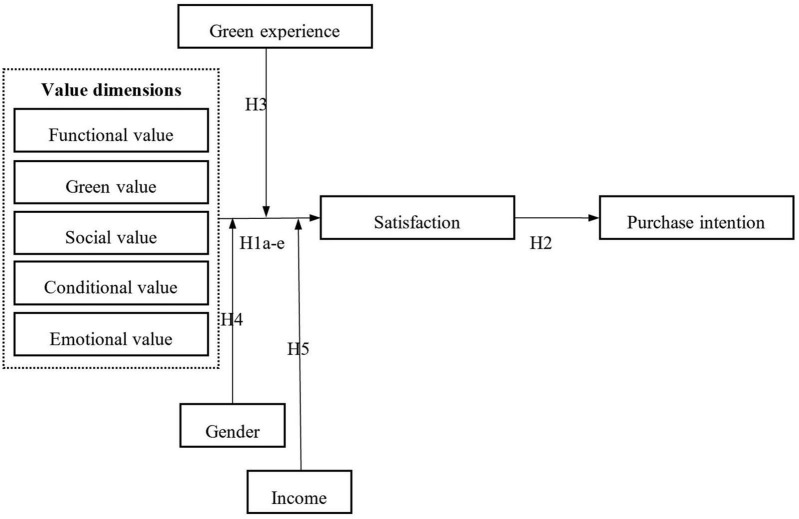
Research model.

Figure 1 exhibits the conceptual model with the proposed relationships.

## Materials and Methods

### Data Collection and Sample

Data are collected online and offline. We first conducted a pilot survey to get important feedback. We initially distributed 50 questionnaires, discussed the feedback, and revised the questionnaires. It is worth noting that according to preliminary research, the time required to complete the questionnaire is usually about 5 min. Then the formal survey was conducted online through the professional online survey website to collect data by random. The sample included consumers who have bought products and consumers who recently wanted to buy energy-saving products. Meanwhile, the questionnaire was distributed at the entrance of a shopping mall in the afternoon, such as SUNING mall, and a gift was given to the participants. Data collection lasted for more than 1 month. According to a *t*-test, there is no significant difference between the data collected online and offline. Thus, the data can be combined for analysis. In total, 455 individuals were invited to complete the questionnaire, of which 422 responded. Of these, 399 surveys were complete and usable, for a response rate of 87.7%. Of the 399 respondents, 144 (36.1%) were potential green customers and 255 (63.9%) were experienced customers. Participant demographics are shown in Table 2.

**TABLE 2:** Consumer characteristics.

Variables	Item	Frequency	Percentage (%)
Gender	Female	178	44.6%
	Male	221	55.4%
Age	Under 20	16	4.0%
	20∼29	344	86.2%
	30∼39	21	5.3%
	40 and above	18	4.5%
Monthly income	Less than 291 dollars	185	46.4%
	$291∼$727	94	23.6%
	$727∼$1,454	83	20.8%
	$1,454 and above	37	9.3%
Education level	High school and secondary specialized school	15	3.8%
	College degree	192	48.1%
	Bachelor and above	192	48.1%
Occupation	Civil servants	17	4.3%
	State workers	34	8.5%
	Non-state workers	43	10.8%
	Teachers	16	4.0%
	Students	219	54.9%
	Others	70	17.6%
Total		399	100%

### Measures and Analysis

The scales of this study are adopted from the extant literature. Functional, social, and conditional value scales are taken from Lin and Huang (2012). A sample item for social value is, “Buying energy-saving products will improve others’ perception of me,” and a sample item for conditional value is, “When there are discounts for energy-saving products, I will buy those products.” The emotional value scale is adopted from Sweeney and Soutar (2001). A sample item is, “Buying energy-saving products will make me feel good.” The green value scale is adapted from Chen (2013). A sample item is, “I buy energy-saving products because they are environmentally friendly.” The customer satisfaction scale is adopted from Suki (2016). A sample item is, “I am satisfied with my decision to purchase the energy-saving products of this company.” The purchase intention scale is adopted from Xu et al. (2019). The wordings were changed to suit this context. A sample item is, “I plan to buy energy-saving products.” The revised questionnaires were sent for pretesting to correct possible defects and doubts. The definition of energy-saving products and specific examples, such as energy-saving lamps and low-energy refrigerators, were provided. The questionnaire was administered in Chinese. We conducted a pretest and received 60 valid responses. A 5-point Likert-type scale was applied to all measurement items, with anchors of 1 = “strongly disagree” and 5 = “strongly agree.”

## Results

### Measurement Reliability and Validity

According to confirmatory factor analysis, the fitting degree of the measurement model was judged. The fitting indexes of measurement model were as follows: χ^2^/df = 2.718, comparative fit index (CFI) = 0.901, parsimonious normed fit index (PNFI) = 0.722, and root mean square error of approximation (RMSEA) = 0.075. It shows that the measurement model fit the data well. Next, this study uses Cronbach’s alpha values and composite reliability to test the reliability of each variable in the questionnaire. Factor loading, internal reliability, convergent validity, and average variance extracted (AVE) were assessed using statistical product and service solutions (SPSS) 20.0. First, the loading items are all greater than 0.50, indicating that structural validity is established. In Table 3, all indicator factor loadings are significant and above 0.50. Second, the Cronbach’s alpha was above 0.70, and the composite reliability was above 0.80, showing that the measurement has high internal reliability. Third, the convergent validity can be evaluated using AVE. AVE exceeds 0.50 (see Table 3), which shows that the scale has high convergent validity. Therefore, it can be considered that convergent validity and internal consistency meet the requirements. Fourth, the square root of AVE of the individual variable was greater than the shared variances of the inter-construct and correlations between the variables, indicating strong discriminant validity (see Table 4). Thus, this study indicates that the measurement model fit the data well.

**TABLE 3:** Reliability and validity analysis.

Constructs	Factor loading	Cronbach’s alpha	Composite reliability	Convergent validity (AVE)
Functional value		0.76	0.85	0.58
FV1	0.51			
FV2	0.60			
FV3	0.63			
FV4	0.59			
Emotional value		0.81	0.88	0.64
EV1	0.59			
EV2	0.63			
EV3	0.75			
EV4	0.59			
Social value		0.85	0.91	0.77
SV1	0.66			
SV2	0.85			
SV3	0.80			
Conditional value		0.86	0.91	0.71
CV1	0.66			
CV2	0.78			
CV3	0.79			
CV4	0.60			
Green value		0.86	0.92	0.78
GV1	0.74			
GV2	0.84			
GV3	0.77			
Consumer satisfaction		0.83	0.89	0.67
CS1	0.73			
CS2	0.49			
CS3	0.73			
CS4	0.74			
Purchase intention		0.81	0.89	0.73
PI1	0.76			
PI2	0.76			
PI3	0.69			

**TABLE 4:** Correlation between variables.

	FV	EV	SV	CV	GV	CS	PI
FV	**0.769**						
EV	0.366**	**0.809**					
SV	0.308**	0.684**	**0.822**				
CV	0.380**	0.573**	0.383**	**0.852**			
GV	0.304**	0.619**	0.470**	0.477**	**0.889**		
CS	0.445**	0.642**	0.471**	0.569**	0.569**	**0.835**	
PI	0.441**	0.593**	0.419**	0.579**	0.579**	0.688**	**0.863**

**Correlation is significant at the 0.01 level (2-tailed); diagonals (in bold) represent the square root of the average variance extracted (AVE).

### Hypothesis Testing

We use the AMOS 21.0 computer program to test our research hypotheses. According to the theoretical model of this study, perceived value is regarded as an exogenous latent variable, customer satisfaction as an intermediary variable, and purchasing intention as an endogenous latent variable. The overall fit index indicates good agreement with the data: the χ^2^ of the model was 522.352 with 251 degrees of freedom (χ^2^/df = 2.081), the CFI was 0.938, the PNFI was 0.743, and the RMSEA was 0.059, indicating a reasonable fit. Therefore, the theoretical model can be used to test the causal path hypothesis.

**FIGURE 2:**
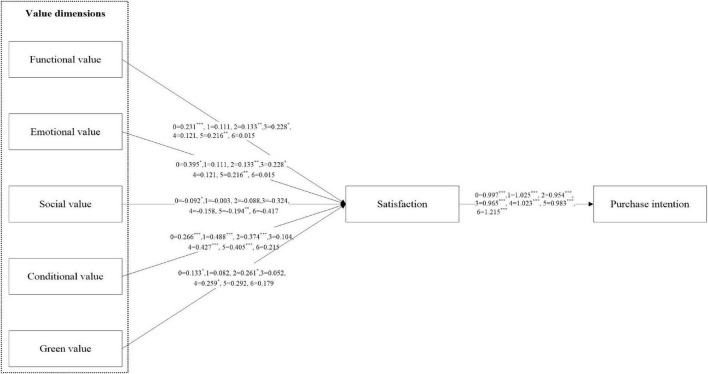
Research results of model. 0, total effect; 1, low green experience; 2, high green experience; 3, male; 4, female; 5, low income; 6, high income.

Taking into account the limitations of the economic-oriented and technology-oriented perspectives, this article aims to promote the purchase of energy-saving products by emphasizing some psychological factors (such as perceived value, satisfaction, etc.) from the psychological-oriented perspective, so as to achieve the goal of green environmental protection. Figure 2 exhibits the research results of model. The main effect is tested by constructing the structural equation model with the results shown in Table 5. The results indicate that functional value has a significant effect on consumer satisfaction (β = 0.231, *p* < 0.01). Emotional value has a significant effect on satisfaction (β = 0.395, *p* < 0.05). Social value is insignificant. Conditional value has a positive effect on consumer satisfaction (β = 0.266, *p* < 0.001). Green value and satisfaction are positively correlated when there is no moderator (β = 0.133, *p* < 0.05). Conditional value has the greatest impact on satisfaction, followed by functional, emotional, and green value. However, only social value is not significant for satisfaction. H1a, H1b, H1d, and H1e are supported. Consumer satisfaction has a highly significant influence on purchasing intention (β = 0.997, *p* < 0.001). Thus, H2 is supported.

**TABLE 5:** Results of the tested hypotheses.

Path	Path coefficient	*t*-value	Hypothesis	Results
Path 1: FV → CS	0.231	2.945**	H1a	Supported
Path 2: EV → CS	0.395	2.368*	H1b	Supported
Path 3: SV → CS	–0.092	–0.851	H1c	N.S.
Path 4: CV → CS	0.266	4.202***	H1d	Supported
Path 5: GV → CS	0.133	2.281*	H1e	Supported
Path 6: CS → PI	0.997	15.064***	H2	Supported

*p < 0.05, **p < 0.01, and ***p < 0.001.

To further test the mediating effect of consumer satisfaction on the relationships between antecedents (i.e., functional, emotional, social, conditional, and green value) and purchasing intention, we performed the mediation test by the PROCESS macro in SPSS 20.0 following the work by Hayes and Preacher (2014). As described in Table 6, the intervals at the 95 percent level of confidence in lines 1, 2, 4, and 5 did not cover 0, which indicated that both the direct and indirect effects associated with functional, emotional, conditional, and green value were significant. However, satisfaction has no mediating effect between social value and purchasing intention following the rule proposed by Baron and Kenny (1986), as social value has an insignificant impact on satisfaction (*p* > 0.05). Therefore, satisfaction plays a mediating role in the influence of perceived value (except social value) on purchasing intention; however, it is not a complete mediating role but a partial mediating role.

**TABLE 6:** Mediation examination results by process macro in SPSS.

IV	Indirect			Direct		
	Effect	LLCI	ULCI	Effect	LLCI	ULCI
FV	0.29	0.19	0.39	0.18	0.08	0.27
EV	0.31	0.23	0.40	0.24	0.14	0.33
CV	0.22	0.16	0.29	0.09	0.02	0.16
GV	0.27	0.20	0.36	0.25	0.16	0.33

*IV, independent variable; LLCI, lower limit of CI; ULCI, upper limit of CI.*

### Multigroup Structural Equation Model ***Analysis***

To further investigate the relationships among variables, multigroup analysis was conducted. The study follows multigroup analysis guidelines (Byrne, 2016). The analysis results are shown in Table 7. It is worth noting that the respondents were divided into two subgroups (low-income and high-income subgroups) based on their monthly income, two subgroups (male and female subgroups) based on gender, and two subgroups (low green experience and high green experience) based on green experience. A low-income subgroup has a monthly income less than $727 and a high-income subgroup has a monthly income greater than $727.

**TABLE 7:** The results of multigroup SEM analysis.

Path	Green experience		Gender		Income	
	Low	High	Male	Female	Low	High
Path 1: FV → CS	0.111	0.133**	0.228*	0.121	0.216**	0.015
Path 2: EV → CS	0.196*	0.391*	0.942*	0.291	0.549***	1.041
Path 3: SV → CS	–0.003	–0.088	–0.324	–0.158	−0.194*	–0.417
Path 4: CV → CS	0.488***	0.374***	0.104	0.427***	0.405***	0.215
Path 5: GV → CS	0.082	0.261**	0.052	0.259*	0.292	0.179
Path 6: CS → PI	1.025***	0.954***	0.965***	1.023***	0.983***	1.215***

*p < 0.05, **p < 0.01, and ***p < 0.001.

In terms of the positive impact of functional value on satisfaction (Path 1), the effect was significant in the high green experience, the male, and the low-income subgroup. In terms of the impact of emotional value on satisfaction (Path 2), the effect was significant in the low and high green experience, the male, and the low-income subgroups. The effects in the high-green experience, the male, and the low-income subgroup were higher than in the other subgroups. In terms of the positive impact of social value on satisfaction (Path 3), the effect was significant in the low-income subgroup. In terms of the positive impact of conditional value on satisfaction (Path 4), the effect was significant in the low and high green experience, the male and female subgroups, and the low-income subgroups. The effects were greater in the low-green experience, the female subgroup, and the low-income subgroup than in the other subgroups. In terms of the positive impact of green value on satisfaction (Path 5), the effect was significant in the high green experience subgroup and the female subgroup. In terms of satisfaction on intentions to purchase energy-saving products (Path 6), the effect was significant in the low and high green experience, the male and female subgroups, and the low-income and high-income subgroups. The effects of satisfaction on intentions to purchase energy-saving products were greater in the low green experience, the female subgroup, and the high-income subgroup than in the other subgroups.

Hypothesis 3 examines the moderating effect of green experience on the relationship between perceived value and satisfaction, that is, the sample was divided into low and high green experience groups. As shown in Table 7, the effect of social value on satisfaction was insignificant and not significantly different between these two groups. Additionally, the path from the green value to satisfaction was higher for the high green experience group than for the low green experience group, and it was significant only for the high green experience group. Only conditional value had a significant effect on satisfaction without green experience. Thus, H3 was partially supported.

Hypothesis 4 examines the moderating effect of gender on the relationship between perceived value and satisfaction, that is, the sample was divided into male and female groups. As shown in Table 7, the effects of social value on satisfaction and emotional value on satisfaction were significantly higher for the male group. Additionally, the effects of conditional value on satisfaction and green value on satisfaction were higher for the female group. The effect of social value on satisfaction was insignificant and not significantly different between these two groups. Thus, H4 was partially supported.

Hypothesis 5 examines the moderating effect of income on the relationship between perceived value and satisfaction, that is, the sample was divided into low- and high-income groups. As shown in Table 7, the effects of functional value on satisfaction, emotional value on satisfaction, social value on satisfaction, and green value on satisfaction were significantly higher for the high green experience group. The effect of green value on satisfaction was insignificant and not significantly different between these two groups. Thus, H5 was partially supported.

## Discussion

This research demonstrated the main influencing factors of perceived value on customer satisfaction and purchasing intention in China. The findings show that consumer satisfaction is positively influenced by functional, emotional, conditional, and green value. Green experience will enhance the overall impact of consumers’ utility perception derived from functional, emotional, conditional, and green value toward green product usage. Therefore, green experience, gender, and income are an important moderator of this relationship.

Given that China is a developing country and many people in China have limited incomes (Wang et al., 2018b), discounts for energy-saving products could significantly influence consumer satisfaction and consumption intention. Moreover, our results substantiate that conditional value has the greatest impact on customer satisfaction, and the effects were greater in the female subgroup, the low-income subgroup, and the subgroup with no green experience are more satisfied with energy-saving products than other subgroups. In addition, the results show that the subgroup with no green experience is more satisfied with energy-saving products care more about the conditional value of products than the subgroup with green experience is more satisfied with energy-saving products. For potential consumers, conditional value is more attractive to potential consumers because what motivates them to try a green product is whether there is a discount on the product. The results indicate that functional value has the second impact on customer satisfaction. High-cost performance attracts consumers (Henkel et al., 2018). If the quality of energy-saving products is good and the function is stable (for example, an energy-saving refrigerator has good cooling effect), the attitude of consumers toward this product is positive (do Paço et al., 2019), which will affect the evaluation of this product. The advanced technology and excellent performance of energy-saving electrical appliances enable consumers to feel satisfied about their use, which will have a positive impact on their purchase attitude (Zhang et al., 2020) and thus affect consumers’ overall evaluation. Emotional value had a positive effect on satisfaction, and the male subgroup, the low-income subgroup, and the subgroup with green experience are more satisfied with energy-saving products have higher effects than other subgroups. In addition, consumers with green experiences are more likely to perceive the emotional value of products than consumers without green experiences.

The results state that an emphasis on enhancing social value does not affect consumer choice behavior. This finding may be because energy-saving appliances belong to individual households (Zhang et al., 2020) and people do not communicate much about others’ appliances. On the other hand, our respondents are mainly young people. They may be self-centered and tend not to be influenced by peer behavior and social norms (Lin and Huang, 2012). Meanwhile, our results also show that green value is the most influential perceived value of energy-saving products relative to other products, and the female subgroup and the subgroup with green experience are more satisfied with energy-saving products have higher effects than other subgroups. For consumers who have previously purchased energy-saving products, perceived green value has a higher influence on satisfaction, which means that consumers who have green experience perceive a high green value of and high satisfaction with energy-saving products.

On the whole, the research shows that consumers with green experience are more significant in the relationship between perceived value and satisfaction than consumers without green experience. Compared with those with no green experience, consumers with green experience are more concerned with environmental issues and with energy-saving products. Because consumers with green experience have purchased green products and are familiar with green products, these consumers have a deep and comprehensive perception of green products (Rashid et al., 2009), which enhances these consumers’ positive emotions about energy-saving products and strengthens the relationship between customer value and satisfaction. Potential consumers are skeptical of the perceived value of energy-saving products and cannot comprehensively evaluate the perceived value of energy-saving products (Zhou and Zhang, 2019).

## Implications, Conclusion, and Limitations

### Implications

This study verified that perceived value has a significant impact on consumers’ purchase intentions of energy-saving products, so the importance of perceived value should be emphasized. With the popularity of green trends worldwide, purchasing intention plays an important role in the field of marketing. If managers try to improve purchase intention, managers of green brands should emphasize value perception because it is the antecedent of behavior intention and the beginning of the contact between consumers and products. Only when consumers have a high assessment of a product can they have an intention to purchase it. The theory of consumption value encourages managers to convey the advantages of products to consumers (Karjaluoto et al., 2012). Thus, managers should be able to grasp the effect of each of the components of consumer perceived value and invest resources to increase this value perception by consumers.

Hypothesis 1a, H1b, H1d, and H1e in the research framework were supported in this study; which means functional value, green value, emotional value, and conditional value significantly and positively influence the purchase intention of energy-saving products. Green marketers need to pay attention to distinguishing the physical and psychological benefits of energy-saving products. First, enterprises should make the physical performance of energy-saving products stable and high; for example, energy-saving air conditioning should offer consumers a good refrigeration effect, and energy-saving lamps enable consumers to achieve stable lighting. Second, although most consumers will not buy energy-saving products simply because of their green attributes (Papista et al., 2018), green marketers should understand that the emphasis on greenness can stimulate green product consumption. Some energy-saving products have seldom been purchased by consumers since marketers have failed to express these products’ green attributes effectively (Hur et al., 2013). Consequently, green marketers should introduce the features of the product comprehensively and emphasize the green value of the product in the process of persuading consumers to buy it. Third, positioning strategies should stress the emotional value provided by the green brand and make good use of their mood state to be pleasant in green consumption. Finally, green marketers should carry out some promotional activities for energy-saving products in a timely manner, which will promote consumers’ attentions and purchase of energy-saving products.

Our study shows that satisfaction has a significant impact on intention to purchase energy-saving products. Satisfied consumers are less sensitive to price and are willing to purchase energy-saving products instead of conventional alternatives (Hur et al., 2013). Therefore, managers can increase consumer satisfaction not only by improving the perceived value of energy-saving products but also by enhancing after-sales service.

Most importantly, manufacturers should pay attention to targeted marketing for different consumer groups because the research has assessed the moderating effect of green experience, gender, and income level on energy-saving products in the perceived value-satisfaction-purchasing intention chain. Through the segmentation of green consumer market, the key green consumers are identified. First, we found the importance of green experience based on the results and this finding informs marketers on how to promote energy-saving products according to the types of consumers. Managers should give potential consumers more opportunities to experience energy-saving products and more preferential policies to encourage these consumers to buy energy-saving products with a pleasant experience, which not only helps the company to make profits and expand its market share but also protects the environment and promotes the social responsibility of the enterprise. Second, to increase consumer satisfaction, measures will be more effective when producers of energy-saving products target consumers with low income.

In addition, the government plays an important role in promoting the development of green products. The government should strengthen quality supervision to ensure the quality of energy-saving products so that consumers who have purchased energy-saving products have a good impression of these products and are satisfied with them. Meanwhile, the government should actively publicize environmental issues, call on consumers to protect the environment, and encourage consumers to purchase environmentally friendly products (Wang et al., 2018a). Finally, the government can provide financial subsidies to manufacturers in order to encourage the development of green product industry.

### Conclusion and Limitations

This study explores the research mechanism between perceived value and purchase intention with the mediating effect of consumer satisfaction with energy-saving products based on the appraisal-emotional response-coping theory in China. Overall, the findings suggest that consumer satisfaction is significantly and positively influenced by four types of the perceived value of energy-saving products (i.e., functional, green, emotional, and conditional value) except social value. In fact, previous scholars have investigated the impact of perceived value on consumers’ intentions to buy energy-saving products. But further researches also need to be conducted due to the changes in energy-saving products industry and market segments, the evolution of consumers. There are some limitations to study the purchase behaviors of energy-saving products from the economic-oriented perspective or technological-oriented perspective, and further researches from the perspective of psychological-oriented perspective make up for the current knowledge gap of energy-saving product consumption.

Furthermore, perceived value is the main driving force of consumers’ purchase intention; our results show that conditional value has the greatest impact on satisfaction, followed by functional, emotional, and green value. When evaluating energy-saving products, consumers must weigh their preferences for the benefits and costs of different products. Green marketers need to pay attention to distinguish the physical and psychological benefits of energy-saving products.

Additionally, the research has assessed the moderating effect of green experience, gender, and income level on energy-saving products in the perceived value-satisfaction-purchasing intention chain. Nowadays, the consumer groups are diversified, so it is necessary to subdivide the market of energy-saving products. It is necessary to make appropriate marketing strategies according to different market segments. Through the segmentation of green consumer market, the key green consumers are identified.

The key direction that should be given priority is to consider the influencing factors and influencing mechanism of consumers’ purchase of energy-saving products from a psychological perspective. In further research, it is necessary to use quantitative techniques to review the literature on energy-saving products, in order to measure the impact of psychological factors on consumers’ choice of energy-saving products.

The limitations of this research are mainly in the following two aspects. First, this research does not specify energy-saving products to a specific product, and each product has its own attributes due to the variety of energy-saving products. Therefore, the perceived value dimension of this study may not be completely applicable to some specific products. Energy-saving products can be subdivided to improve the related research in the future. Second, this research studies the influencing factors of purchase intention. However, there is still a gap between intention and actual behavior, although intention affects actual behavior immediately. Hence, future researches can focus on the purchase behavior of energy-saving products rather than purchase intentions of energy-saving products.

## Data Availability Statement

The raw data supporting the conclusions of this article will be made available by the authors, without undue reservation.

## Author Contributions

BL and YS contributed to research design. LL conducted to the sample collection and data analysis and wrote the manuscript. YS did text review and editing. All authors contributed to the article and approved the submitted version.

## Conflict of Interest

The authors declare that the research was conducted in the absence of any commercial or financial relationships that could be construed as a potential conflict of interest.

## Publisher’s Note

All claims expressed in this article are solely those of the authors and do not necessarily represent those of their affiliated organizations, or those of the publisher, the editors and the reviewers. Any product that may be evaluated in this article, or claim that may be made by its manufacturer, is not guaranteed or endorsed by the publisher.
